# Child somatic growth and neurodevelopment: effects of pregnancy lifestyle intervention

**DOI:** 10.1038/s41390-025-03936-y

**Published:** 2025-02-20

**Authors:** Kristina Geyer, Roxana Raab, Monika Spies, Johanna Knoke, Dorothy Meyer, Stephanie Brandt-Heunemann, Hans Hauner

**Affiliations:** 1 Institute of Nutritional Medicine, Else Kröner-Fresenius-Centre for Nutritional Medicine, TUM School of Medicine and Health, Technical University of Munich, Georg-Brauchle-Ring 62, 80992 Munich, Germany; 2 Centre for Hormonal Disorders in Children and Adolescents, Division of Paediatric Endocrinology and Diabetes, Department of Paediatrics and Adolescent Medicine, Ulm University Medical Centre, Eythstraße 24, 89075 Ulm, Germany

## Abstract

**Objective:**

Maternal health behavior influences offspring health and obesity risk. This study examined the long-term effects of an antenatal lifestyle intervention on somatic growth and neurodevelopment of preschool-aged children.

**Methods:**

We followed children born to women in the cluster-randomized GeliS trial who received usual care (CG) or lifestyle counseling (IG). Anthropometrics and neurodevelopment data for children aged 4 and 5 were collected from routine health examinations and the Ages-and-Stages Questionnaire (ASQ).

**Results:**

Of 2 286 women initially enrolled, 1 403 reported on their child’s development. The intervention had no effect on weight, height, head circumference, BMI, or percentiles and z-scores at ages 4 and 5. In IG compared to CG, the proportion of children with underweight was lower (4 years: 7.8% vs. 10.9%; 5 years: 8.1% vs. 8.9%), while overweight (4 years: 6.5% vs. 4.2%; 5 years: 5.1% vs. 3.4%) and obesity proportions (4 years: 1.0% vs. 1.1%; 5 years: 2.7% vs. 1.6%) were higher. IG children were more likely to fall into a higher weight category at 4 (*p* = 0.017) and 5 years (*p* = 0.075). ASQ scores were similar across both groups.

**Conclusion:**

Despite slight weight differences, the pregnancy lifestyle intervention had no meaningful impact on child somatic growth or neurodevelopment up to age 5.

**Impact:**

- This comprehensive antenatal lifestyle intervention, executed as a large-scale real-world effectiveness trial, did not demonstrate any long-term effect on children’s anthropometry or their risk of overweight or obesity up to 5 years of age.
- No discernible intervention effects were observed concerning children’s neurodevelopment outcomes.
- Personalized antenatal interventions targeting the individual risk profiles of pregnant women may be needed to substantially modify lifestyle behaviors and achieve sustainable impacts on child development and obesity risk.

## Introduction

Childhood overweight and obesity is a complex, multifactorial disease that is associated with numerous adverse health outcomes. These include an increased risk of elevated blood pressure, lipid or glucose metabolism disorders, type 2 diabetes, cardiovascular disorders, and cancer later in life.^1^ In addition, children with overweight and obesity suffer a notable decline in quality of life^2^ and are more likely to face bullying.^3^

The causes of overweight in childhood are diverse, with early life exposures playing a key role, particularly during prenatal and postnatal developmental stages. For instance, altered intrauterine conditions, influenced by maternal overweight, obesity and excessive gestational weight gain (GWG)^4,5^ can lead to epigenetic changes in fetal metabolism. These changes have been shown to elevate the risk of high birth weight, large for gestational age, rapid infant weight gain, and overweight and obesity later in life.^6,7^ Additionally, maternal overweight and obesity may impair offspring neuroendocrine regulation, neuronal pathways, and brain structure due to intrauterine exposure to an excess of circulating nutrients as well as elevated levels of certain hormones and inflammatory cytokines. It is posited that these factors may contribute to later neurobehavioral problems.^8,9^ On the other hand, adhering to a healthy lifestyle during pregnancy, along with a healthy pre-pregnancy body mass index (BMI)^10^ and breastfeeding,^11^ has been linked to lower childhood obesity risk. Further, optimizing nutrition during fetal and early postnatal stages can enhance child neurodevelopment and brain function.^12^

Recent research has focused on interventions aimed at disrupting the transmission of intergenerational obesity and promoting healthy child development.^13,14^ In the GeliS (“healthy living in pregnancy”) trial, pregnant women in the intervention group (IG) received lifestyle counseling compared to standard care in the control group (CG).^15^ While the IG women improved some health behaviors,^16–18^ it did not affect the proportion of women with excessive GWG.^19^ Follow-up analyses on the children of GeliS participants found no intervention effects on anthropometry and neurodevelopment until three years of age.^20,21^ In this analysis, we examine whether the GeliS intervention conferred any long-term effects on children’s somatic growth and neurodevelopment at 4 and 5 years of age, hypothesizing that the IG will show lower prevalence of overweight and obesity and better neurodevelopmental outcomes.

## Subjects and methods

### Design and setting of the GeliS study

The GeliS study is a prospective, cluster-randomized, controlled, open intervention trial that integrated lifestyle counseling alongside standard German antenatal care. The trial was carried out across ten regions within Bavaria, Germany, that were randomly assigned as either intervention or control areas. Detailed information on the study design and cluster-randomization can be found in the published study protocol.^15^ The primary aim was to decrease the proportion of women who gained excessive gestational weight according to the thresholds established by the National Academy of Medicine^22^ and to decrease the incidence of gestational diabetes mellitus. Results on primary and secondary outcomes are published elsewhere.^16–21,23^ The study adhered to local regulatory standards and followed the principles outlined in the Declaration of Helsinki. The study protocol was approved by the Technical University of Munich Ethics Committee (project number 5653/13), and was registered in the ClinicalTrials.gov Protocol Registration System (NCT01958307).

### Participants and lifestyle intervention program

A total of 71 gynecological and midwifery practices actively participated in the recruitment of eligible pregnant women from 2013 to 2015. Inclusion criteria comprised the following: having a pre-pregnancy BMI ranging from ≥ 18.5 kg/m² to ≤ 40.0 kg/m², aged between 18 and 43 years, gestational age less than the 12^th^ week, singleton pregnancy, proficiency in German, and provision of informed consent. Women experiencing severe complications during pregnancy were excluded from the study.^15^ During the follow-up, women were characterized as drop-outs if they failed to provide contact details, became unreachable, or withdrew from participation.^23^

Participants allocated to the IG underwent an antenatal lifestyle intervention program, which consisted of four face-to-face counseling sessions provided in the participating practices. These sessions were integrated into routine antenatal care visits and were delivered by medical personnel, midwives, or gynecologists who received prior training on study materials by the research staff. Three counseling sessions were scheduled during pregnancy (12^th^–16^th^, 16^th^–20^th^, 30^th^–34^th^ week of gestation), and were 30 to 45 min in duration. Additionally, a counseling session was conducted at 6–8 weeks postpartum.

The counseling addressed various topics, including adherence to appropriate GWG according to the National Academy of Medicine recommendations,^22^ guidance on maintaining a healthy diet and engaging in physical activity based on national and international recommendations,^24,25^ the importance of a healthy antenatal and postnatal lifestyle, the benefits of breastfeeding over formula, and supportive information on introducing complementary feeding. A comprehensive overview of the lifestyle intervention program and counseling content is available in the study protocol.^15^ Women in the CG received routine antenatal care with the addition of an informational flyer containing general guidance on maintaining a healthy lifestyle during pregnancy and lactation. Both CG and IG participants were enrolled in a 5-year follow-up observation program, during which GeliS researchers collected data on mother-child pairs through phone interviews and mailed questionnaires at 1, 3, and 5 years after childbirth.

### Collection and processing of data

Maternal sociodemographic data was obtained through a screening questionnaire, administered at recruitment before the 12^th^ week of gestation. Pre-pregnancy BMI was calculated using self-reported pre-pregnancy weight and height. Maternal weight during pregnancy was derived from maternity records. GWG was determined by calculating the difference between the last measured weight before delivery and the initial measured weight at recruitment. Information on the current smoking status of mothers was gathered from a questionnaire completed by participants five years after giving birth.

Birth date and anthropometric measurements were obtained from birth records. The child’s age was calculated by subtracting the date of birth from the respective data collection time point, and single imputation was applied in cases where questionnaire completion dates were missing.

Well-child health visits are planned between 46–48 months and 60–64 months of life as part of routine pediatric health examinations in Germany. Anthropometric data collected during these visits is recorded in a check-up booklet. The data were provided by participants during phone interviews around the child’s 5^th^ birthday and were used to calculate age- and sex-specific percentiles and z-scores based on a German reference group.^26^ BMI-for-age percentiles below 10.0 classified children as having underweight, percentiles above 90.0 as having overweight, and percentiles above 97.0 as having obesity.^26^

Neurodevelopmental data for 5 year-old children was collected with the German 60-month version of the validated Ages and Stages Questionnaire (ASQ-3^TM^), which was mailed to participants for them to complete independently.^27^ The ASQ consists of five developmental domains: communication, gross motor skills, fine motor skills, problem-solving and personal-social development. Each domain comprises six questions focusing on age-appropriate development. Answers are given numerical values according to the following: “yes” responses ( = 10 points) indicate that the child has mastered the task, “sometimes” ( = 5 points) means that the task is not frequently mastered, or “not yet” ( = 0 points) means that the child has not mastered the task. For domains with two or fewer missing responses, the mean value of the remaining responses was computed. If three or more responses were missing, no score was given for that domain and was excluded from analysis. Each of the domains was scored separately and scores were tallied and compared to unique cut-off scores between 0 and 60. Scores above the cut-off values indicated that the child’s development was on target for their age, whereas scores below cut-off values suggest a potential developmental delay in the corresponding domain. In accordance with the user’s guide,^27^ the following cutoff scores are assigned for each domain: 33.19 for communication, 31.28 for gross motor skills, 26.54 for fine motor skills, 29.99 for problem-solving, and 39.07 for personal-social development.^27^

### Statistical analysis

The power calculation for the GeliS study was based on the primary endpoint of excessive GWG.^15^ This study describes findings from intention-to-treat secondary analyses and included all participants who provided child anthropometric and/or neurodevelopment data. Between-group differences in child anthropometric values were computed using likelihood-based mixed models for repeated measures, as described by Bell et al.^28^ Data from all well-child health visits, conducted annually between the ages of 1 and 5 years, was included. Customized hypotheses were applied to investigate group differences at 4 and 5 years old. Point estimates and 95% confidence intervals (CI) for the mean differences between the IG and CG at 4 and 5 years of age were calculated in models that included visit number, group assignments, and their interaction terms. These models were adjusted for study region as well as various modifiable and non-modifiable factors based on previously observed group differences,^20,21,29^ including maternal pre-pregnancy age, maternal pre-pregnancy BMI category, parity and child sex. Additionally, child age in days at the respective visit was an adjustment covariate to account for deviations from the suggested visit time point. Weight categories (i.e. underweight, normal weight, overweight and obesity) were determined by BMI-for-age percentiles at 4 and 5 years of age. Between-group differences were investigated using proportional odds ordinal logistic regression models. These models were constructed using generalized estimating equations, as per the approach outlined by Donner et al.^30^ The analysis was adjusted for maternal pre-pregnancy age, pre-pregnancy BMI category, and parity. Analyses of age- and sex-specific percentiles and z-scores were not adjusted for the sex and age of the child.

Differences in ASQ scores were examined through linear regression models, employing generalized estimating equations. These models were also adjusted for maternal pre-pregnancy age, maternal pre-pregnancy BMI category, parity and child sex. Adjustments for child age in months at completion were incorporated to accommodate deviations from the intended age range for the 60-month version of the ASQ. Additionally, maternal educational level was included as an adjustment covariate for consistency with both the previous GeliS publication on neurodevelopment^21^ and other randomized controlled trials (RCTs) in the research field.^31^ Binary logistic regression models utilizing generalized estimating equations were applied to explore group differences regarding the proportion of infants with ASQ scores falling below cut-off values. These models were unadjusted due to the small number of observations.

All analyses were performed using SPSS software (IBM SPSS Statistics for Windows, version 29.0, IBM Corp, Armonk, NY), and statistical significance was defined as two-tailed *p* values < 0.05. Given the exploratory character of the analyses, no corrections for multiple testing were implemented.

## Results

### The flow of GeliS participants

**Fig. 1:**
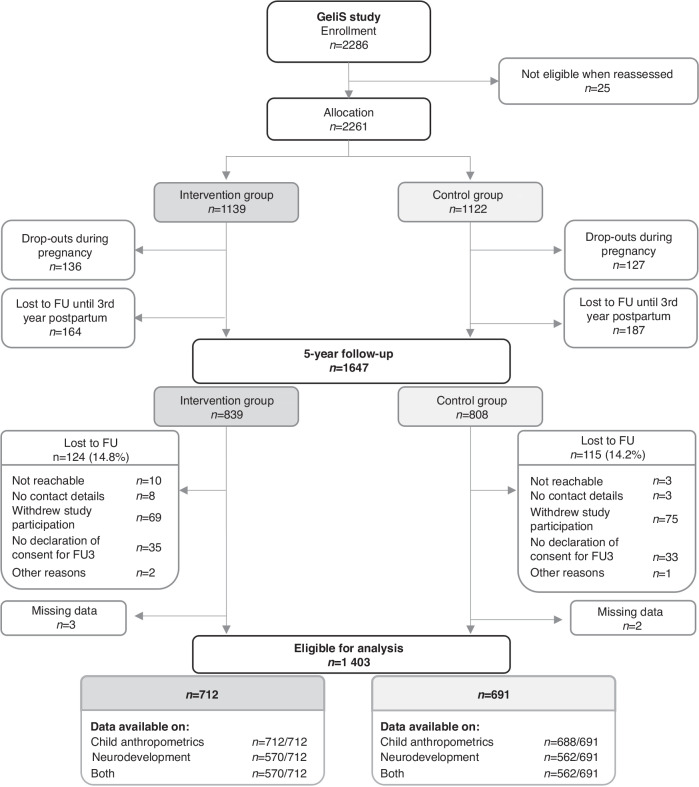
Flow of GeliS participants. GeliS Gesund leben in der Schwangerschaft/healthy living in pregnancy, FU Follow-up.

From the initially recruited 2 286 women for the GeliS study, 1647 women were included in the 5-year follow-up. For 124 women from the IG and 116 women from the CG, loss to follow-up occurred for various reasons. In five participants, information was missing, resulting in the 1403 mother-child pairs eligible for analyses of anthropometric and neurodevelopment data of the children (Fig. 1).

### Baseline characteristics of mother-child pairs

Table 1 presents the characteristics of the mother-child pairs considered in the present analysis. In total, 67.0% of women began their pregnancy with normal weight, 22.3% with overweight, and 10.7% with obesity. Maternal pre-pregnancy age, weight, BMI, and GWG were similar between the IG and CG women. The percentage of primipara women was higher in the IG compared to the CG (65.0% vs. 55.6%, *p* = 0.004), which was the only statistically significant difference in maternal baseline characteristics (Table S1).

**Table 1:** Characteristics of mother-child pairs.

	Intervention group *n* = 712	Control group *n* = 691	Total *n* = 1403
*Maternal characteristics*			
Pre-pregnancy age, years^a^	30.6 ± 4.1	30.8 ± 4.3	30.7 ± 4.2
Pre-pregnancy weight, kg	67.9 ± 12.7	67.4 ± 13.1	67.6 ± 12.9
Pre-pregnancy BMI, kg/m^2^	24.2 ± 4.2	24.0 ± 4.5	24.1 ± 4.3
Pre-pregnancy BMI category, *n* (%)			
BMI 18.5–24.9 kg/m^2^	467/712 (65.6%)	473/691 (68.5%)	940/1403 (67.0%)
BMI 25.0–29.9 kg/m^2^	170/712 (23.9%)	143/691 (20.7%)	313/1403 (22.3%)
BMI 30.0–40.0 kg/m^2^	75/712 (10.5%)	75/691 (10.9%)	150/1403 (10.7%)
GWG, kg	13.8 ± 5.2	14.0 ± 5.0	13.9 ± 5.1
GDM, *n* (%)	72/700 (10.3%)	56/653 (8.6%)	128/1353 (9.5%)
Educational level, *n* (%)^b^			
General secondary school	77/711 (10.8%)	96/690 (13.9%)	173/1401 (12.3%)
Intermediate secondary school	307/711 (43.2%)	282/690 (40.9%)	589/1401 (42.0%)
High school	327/711 (46.0%)	312/690 (45.2%)	639/1401 (45.5%)
Country of birth, *n* (%)			
Germany	640/712 (89.9%)	628/690 (91.0%)	1268/1402 (90.4%)
Others	72/712 (10.1%)	62/690 (9.0%)	134/1402 (9.6%)
Primiparous, *n* (%)	463/712 (65.0%)	384/691 (55.6%)	847/1403 (60.4%)
Current smoker, *n* (%)^c^	93/645 (14.4%)	75/612 (12.3%)	168/1257 (13.4%)
*Infant characteristics at birth*			
Sex, *n* (%)			
Male	360/712 (50.6%)	369/691 (53.4%)	729/1403 (52.0%)
Female	352/712 (49.4%)	322/691 (46.6%)	674/1403 (48.0%)
Preterm birth, *n* (%)	43/709 (6.1%)	42/691 (6.1%)	85/1400 (6.1%)
SGA, *n* (%)	60/709 (8.5%)	51/691 (7.4%)	111/1400 (7.9%)
LGA, *n* (%)	55/709 (7.8%)	53/691 (7.7%)	108/1400 (7.7%)
Birth weight > 4000 g, *n* (%)	62/711 (8.7%)	58/691 (8.4%)	120/1402 (8.6%)

*BMI* body mass index, *GWG* gestational weight gain, *GDM* gestational diabetes mellitus, *SGA* small for gestational age ( < 10^th^ percentile); *LGA* large for gestational age ( > 90^th^ percentile); *SD* standard deviation.

^a^Mean ± SD (all such values).

^b^General secondary school: General school, which is completed through year 9; Intermediate secondary school: Vocational secondary school, which is completed through year 10; High school: Academic high school, which is completed through year 12 or 13.

^c^Collected five years after birth.

Adverse pregnancy outcomes, such as preterm birth, small for gestational age, large for gestational age, and high birth weight, were largely comparable between the groups. The proportions of mother-infant pairs lost to follow-up were also similar (Table S2). However, characteristics of women lost to follow-up differed slightly from those who remained, such as pre-pregnancy weight, GWG, education, country of birth, and parity. Nonetheless, infant birth outcomes were comparable between those groups (Table S2).

### Child anthropometrics

Table 2 presents the child anthropometric data at 4 and 5 years of age. At 4 years old, a statistically significant group difference was observed in weight percentiles (adj. effect size 2.99, 95% CI 0.18 to 5.80, *p* = 0.037) between IG and CG, but not in mean weight (adj. effect size 0.19, 95% CI −0.03 to 0.40, *p* = 0.091) or weight z-scores (adj. effect size 0.09, 95% CI 0.00 to 0.19, *p* = 0.053). At 5 years of age, there were significant group differences in mean weight (adj. effect size 0.33, 95% CI 0.04 to 0.61, *p* = 0.023), weight z-scores (adj. effect size 0.12, 95% CI 0.02 to 0.21, *p* = 0.021), and weight percentiles (adj. effect size 3.49, 95% CI 0.62 to 6.36, *p* = 0.017). Mean height, respective sex- and age-specific outcomes, and mean head circumference at both ages were similar between groups. At 5 years old, there were significant between-group differences in mean BMI (adj. effect size 0.19, 95% CI 0.04 to 0.34, *p* = 0.013), BMI z-scores (adj. effect size 0.12, 95% CI 0.02 to 0.21, *p* = 0.015), and BMI percentiles (adj. effect size 3.39, 95% CI 0.57 to 6.21, *p* = 0.018).

**Table 2:** Child anthropometrics at 4 and 5 years of age.

	Age	Intervention group		Control group		Adjusted effect size^a^ (95% CI)	Adjusted *p* value^a^
		*n*	Mean ± SD	*n*	Mean ± SD		
Weight, kg	4 years	696	16.82 ± 2.08	664	16.63 ± 2.08	0.19 (−0.03, 0.40)^b^	0.091
	5 years	708	19.61 ± 2.78	684	19.32 ± 2.65	0.33 (0.04, 0.61)	0.023
Height, cm	4 years	696	103.97 ± 4.06	662	103.72 ± 4.10	0.30 (−0.12, 0.72)	0.165
	5 years	708	112.40 ± 4.62	684	112.21 ± 4.64	0.34 (−0.14, 0.81)	0.163
Head circumference, cm	4 years	488	50.51 ± 1.51	425	50.52 ± 1.43	0.05 (−0.11, 0.21)	0.537
	5 years	479	51.36 ± 1.52	397	51.22 ± 1.42	0.16 (−0.01, 0.32)	0.060
BMI, kg/m^2^	4 years	695	15.52 ± 1.31	662	15.42 ± 1.31	0.12 (−0.02, 0.26)	0.094
	5 years	708	15.47 ± 1.48	683	15.30 ± 1.40	0.19 (0.04, 0.34)	0.013
Weight z-score ^c^	4 years	696	0.05 ± 0.89	664	−0.04 ± 0.89	0.09 (0.00, 0.19)	0.053
	5 years	708	−0.03 ± 0.94	684	−0.14 ± 0.92	0.12 (0.02, 0.21)	0.021
Weight percentile ^c^	4 years	696	51.47 ± 26.63	664	48.79 ± 26.84	2.99 (0.18, 5.80)	0.037
	5 years	708	49.08 ± 27.27	684	45.89 ± 27.32	3.49 (0.62, 6.36)	0.017
Height z-score	4 years	696	0.08 ± 0.92	662	0.03 ± 0.91	0.06 (−0.04, 0.16)	0.231
	5 years	708	0.02 ± 0.93	684	−0.03 ± 0.92	0.06 (−0.04, 0.16)	0.255
Height percentile	4 years	696	52.46 ± 27.21	662	50.55 ± 27.23	2.05 (−0.83, 4.94)	0.163
	5 years	708	50.43 ± 27.51	684	48.59 ± 27.65	2.09 (−0.83, 5.01)	0.160
BMI z-score	4 years	695	−0.01 ± 0.89	662	−0.08 ± 0.91	0.08 (−0.01, 0.18)	0.088
	5 years	708	−0.04 ± 0.91	683	−0.15 ± 0.90	0.12 (0.02, 0.21)	0.015
BMI percentile	4 years	695	49.78 ± 27.18	662	48.01 ± 26.98	2.19 (−0.65, 5.04)	0.131
	5 years	708	48.43 ± 27.22	683	45.23 ± 26.79	3.39 (0.57, 6.21)	0.018
**Weight category**	4 years	***n*** **(%)**		***n*** **(%)**		**Adjusted odds ratio** ^**d**^ **(95% CI)**	**Adjusted** ***p*** **value** ^**d**^
Underweight < 10^th^ BMI percentile		54/695 (7.8%)		72/662 (10.9%)		1.51 (1.08, 2.12)	0.017
Normal weight 10^th^ - 90^th^ BMI percentile		589/695 (84.7%)		555/662 (83.8%)			
Overweight 90^th^ - 97^th^ BMI percentile		45/695 (6.5%)		28/662 (4.2%)			
Obesity (reference category) > 97^th^ BMI percentile		7/695 (1.0%)		7/662 (1.1%)			
**Weight category**	5 years	***n*** **(%)**		***n*** **(%)**			
Underweight < 10^th^ BMI percentile		57/708 (8.1%)		61/683 (8.9%)		1.25 (0.98, 1.59)	0.075
Normal weight 10^th^ - 90^th^ BMI percentile		596/708 (84.2%)		588/683 (86.1%)			
Overweight > 90^th^ - 97^th^ BMI percentile		36/708 (5.1%)		23/683 (3.4%)			
Obesity (reference category) > 97^th^ BMI percentile		19/708 (2.7%)		11/683 (1.6%)			

*CI* confidence interval, *SD* standard deviation, *BMI* body mass index.

^a^From mixed models for repeated measures with the use of data from each health examination from the 1^st^ to the 5^th^ year of life and controlled for maternal pre-pregnancy age, maternal pre-pregnancy BMI category, parity, child sex, child age in days at the corresponding visit and study region, except for age- and sex-specific percentiles and z-scores, which were not adjusted for child age and sex.

^b^Estimated mean difference; in parentheses 95% CI (all such values).

^c^All z-scores and percentiles were calculated according to Kromeyer-Hauschild et al.^26^.

^d^From proportional odds ordinal logistic regression models fit using generalized estimating equations adjusted for maternal pre-pregnancy age, maternal pre-pregnancy BMI category and parity.

The prevalence rates for underweight, normal weight, overweight and obesity among 4-year-old children were 9.4%, 84.3%, 5.4% and 1.1%, respectively. At 5 years of age, these rates were 8.5%, 85.2%, 4.3% and 2.2%. The odds for being in a higher weight category were significantly different for children in the IG compared to the CG at 4 years old (adj. OR 1.51, 95% CI 1.08 to 2.12, *p* = 0.017), however, this difference was no longer significant at 5 years old (adj. OR 1.25, 95% CI 0.98 to 1.59, *p* = 0.075) (Table 2).

### Child neurodevelopment

Table 3 shows the ASQ scores for the five different domains for 5-year-old children. Communication (*p* = 0.143), gross motor skills (*p* = 0.627), fine motor skills (*p* = 0.523), problem-solving (*p* = 0.628), and personal-social development (*p* = 0.383) scores were comparable in both groups with no statistical group differences.

**Table 3:** Child neurodevelopment assessed by ASQ scores in the five domains at 5 years of age.

	Intervention group		Control group		Adjusted effect size^a^ (95% CI)	Adjusted *p* value^a^
	*n*	Mean ± SD	*n*	Mean ± SD		
Communication	567	54.27 ± 7.13	562	54.57 ± 6.89	−0.42 (−0.99, 0.14)	0.143
Gross motor	567	56.03 ± 6.21	565	56.26 ± 6.31	−0.22 (−1.12, 0.67)	0.627
Fine motor	555	51.75 ± 9.76	542	52.16 ± 9.38	−0.43 (−1.77, 0.90)	0.523
Problem-solving	563	54.72 ± 6.52	560	54.71 ± 6.64	−0.14 (−0.73, 0.44)	0.628
Personal-social	565	57.12 ± 4.63	563	57.41 ± 4.33	−0.24 (−0.78, 0.30)	0.383

*ASQ* Ages and Stages Questionnaire (ASQ-3TM), *SD* standard deviation, *CI* confidence interval.

^a^Linear regression model fit using generalized estimating equations adjusted for maternal pre-pregnancy age, maternal pre-pregnancy BMI category, maternal educational level, parity, child sex, and child age in months at completion of the ASQ.

The percentage of children in both groups achieving scores below the cut-off values in communication (IG 1.8% vs. CG 1.6%, *p* = 0.912), gross motor skills (IG 0.7% vs. CG 1.1%, *p* = 0.531), fine motor skills (IG 2.9% vs. CG 3.1%, *p* = 0.767), problem-solving (IG 0.4% vs. CG 0.2%, *p* = 0.873) and personal-social development (IG 0.5% vs. CG 0.4%, *p* = 0.699) were similar, with no evidence of intervention effects in unadjusted models (Table 4).

**Table 4:** Proportion of children with ASQ scores below cut-off at 5 years of age.

	Intervention group		Control group		Unadjusted odds ratio^a^ (95% CI)	Unadjusted *p* value^a^
	*n*	(%)	*n*	(%)		
Communication	10/567	1.8%	9/562	1.6%	0.97 (0.52, 1.78)	0.912
Gross motor	4/567	0.7%	6/565	1.1%	0.60 (0.12, 2.97)	0.531
Fine motor	16/555	2.9%	17/542	3.1%	0.90 (0.44, 1.84)	0.767
Problem-solving	2/563	0.4%	1/560	0.2%	1.09 (0.37, 3.22)	0.873
Personal-social	3/565	0.5%	2/563	0.4%	1.47 (0.21, 10.46)	0.699

Depicted is the proportion of children from the IG and CG with ASQ scores below the cut-off in the individual domains. *ASQ* Ages and Stages Questionnaire (ASQ-3TM), *CI* confidence interval.

^a^Unadjusted binary logistic regression model fit using generalized estimating equations.

## Discussion

The aim of this study was to assess whether somatic growth and neurodevelopment differed between children whose mothers participated in an antenatal lifestyle intervention compared to those who received standard antenatal care. Our results showed that children in the IG had significantly higher odds of being in a higher weight category at 4 years old compared to the CG. This finding was also observed at 3 years old, as reported by Spies et al.^21^ At 5 years old, IG children continued to show higher somatic growth, with statistically significant higher weight and BMI outcomes. In fact, these children showed consistently lower rates of underweight and higher rates of overweight and obesity compared to the CG. In this analysis we observed no appreciable differences in domain-specific ASQ scores between IG and CG children at 4 and 5 years old. These results contrast with earlier findings, which showed minor between-group differences at 3 years old.^21^ In both groups, the proportion of children scoring below the cut-off in any domain, indicating a risk for neurodevelopmental delays, was very low and ranged from 0.2–3.1%. To summarize, earlier GeliS follow-up assessments of children from birth to 3 years showed no intervention effect on the prevalence of overweight or obesity, nor on neurodevelopment outcomes.^20,21^ This current analysis further supports the lack of meaningful clinical impact on somatic growth and neurodevelopment in preschool-aged children. Our hypothesis that the gestational period represents an opportunity for interventions to reduce the risk of childhood obesity and positively influence neurodevelopment could not be supported by our findings.

One possible reason for the GeliS lifestyle program’s lack of effectiveness on long-term childhood outcomes is its failure to reduce the proportion of women who gained excessive gestational weight.^19^ Despite following the GeliS program curriculum, it is very likely that the healthcare practitioners responsible for counseling women used different approaches and varied in the information they provided. While the method of counseling was inherently part of the public health aspect of the GeliS study, these inconsistencies may have reduced the program’s overall effectiveness.^19^ Although we observed moderate improvements in maternal dietary habits and physical activity,^16–18^ these did not result in significant improvements on long-term somatic growth and neurodevelopment of the offspring. Since an elevated risk of childhood adiposity increases with higher maternal GWG,^5^ more effective interventions aimed at reducing GWG might have the potential to positively impact childhood growth and obesity risk.

Previous systematic reviews have reported that antenatal lifestyle interventions are associated with reduced GWG,^32,33^ but observed group differences are likely too modest to have a clinically significant impact on outcomes for both women and children. Our findings are consistent with recent systematic reviews that examined antenatal intervention trials which were conducted in various countries and utilized different intervention modalities and intensities. For instance, Raab et al.^14^ found no sustained effects of antenatal lifestyle interventions on offspring anthropometry from 1 month to 7 years of age. This lack of positive impact was consistent in subgroup analyses examining intervention content and duration. Similarly, an individual participant data meta-analysis on antenatal lifestyle interventions for high-risk populations of women with overweight and obesity found no effects on infant anthropometry nor neurodevelopment outcomes at 3 to 5 years of age.^13^

Although most GeliS participants began their pregnancies in the normal weight range, similar negative findings have also been reported in RCTs focusing on women with overweight and/or obesity. For example, the Australian LIMIT trial showed no effect on child growth, adiposity, or neurodevelopment, at 6 months,^34^ 18 months^35^ and 3 to 5 years of age.^36^ Long term follow up of this cohort continued to show no effect on childhood obesity risk or secondary developmental measures at 8 to 10 years of age.^37^ Similar results were reported from the UPBEAT trial^38^ which demonstrated no lasting impact on child growth or adiposity, despite the relatively intense prenatal intervention method. The overall lack of positive effects suggests that postnatal environmental factors may have a stronger influence on weight and adipose tissue development, thereby overriding early prenatal influences.^39,40^

Two smaller trials have further explored the impact of lifestyle interventions on child neurodevelopment, with mixed results. Menting et al.^41^ found that two RCTs conducted in women during the preconception and prenatal periods resulted in no significant improvements on neurodevelopment in a subsample of children aged 3 to 6 years. In contrast, Braeken and Bogaerts^31^ reported that children whose mothers participated in a brochure-based lifestyle intervention had improved temperament at 3 to 7 years, compared to those whose mothers received routine antenatal care along with additional lifestyle intervention sessions. Overall, the comparability of results is complicated by the fact that studies recruit diverse study populations, take place in various settings, and use a variety of measurement tools, which limits the ability to draw conclusions. Moreover, the lack of consistency in how researchers measure obesity is problematic and has recently been addressed in a systematic review advocating for the agreement of a core outcome set.^42^ In summary, it appears unlikely that current lifestyle interventions in pregnancy have a long-lasting effect on children’s somatic growth or neurodevelopment. Further research is needed to explore whether more holistic and personalized approaches in healthcare settings can produce lasting effects on child development and obesity risk. This effort should most likely begin earlier and focus on interventions that seek to optimize women’s health and weight before conception, due to the strong link between maternal pre-pregnancy BMI and offspring adiposity. Another approach is exploring how technology can enhance the efficiency and impact of interventions for child health.^43^ For instance, mobile application-supported adaptive interventions, including tele-coaching, may offer a promising way to facilitate changes in maternal lifestyle behaviors. Additionally, tailored interventions based on rigorous early pregnancy risk assessment,^44^ and involving both parents or the entire family^45,46^ show potential for improving outcomes. Finally, robust public health policies are crucial, acknowledging the role that environmental factors have on the health of individuals, families, and communities.

The follow-up analysis has several strengths worth mentioning. The GeliS trial is one of the largest RCTs worldwide evaluating a comprehensive lifestyle intervention conducted within the framework of routine antenatal care. Including pregnant women from different BMI categories broadens the applicability of findings. The long-term follow-up for mother-child pairs at 1, 3 and 5 years gave us access to data on weight, length and head circumference throughout early childhood. Thus, this paper extends our understanding by addressing data up to preschool age, a period critical for adiposity rebound which can predict later obesity.^47,48^ A particular strength of the study is that more than two thirds of participants from the original GeliS cohort provided full datasets of their children at 5 years of age. This signifies an exceptionally high retention rate during the follow-up phase in comparison to other lifestyle intervention trials.^38^

Despite the findings presented, it is important to consider several limitations. First, we observed differences in some characteristics between mothers who declined participation during the five-year follow-up and those who continued (Table S2). While statistical models adjusted for some of these characteristics, we cannot completely rule out the possibility of confounding. Additionally, child height, weight and head circumference were measured by clinic staff during routine visits, and lack of standardization may reduce comparability. A further limitation was that part of the follow-up period coincided with the COVID-19 pandemic, which led to delays in well-child health visits for 52% of GeliS children, impacting the timing of anthropometric measurements at 5 years old. The ASQ was mailed to participants and completed independently, resulting in delays as well as variations in children’s ages when the ASQ was filled out. To account for these timing discrepancies, we calculated the actual age of the children at each data point collection time and included them as covariates in the analyses. Lastly, we used German-based reference values for z-scores and weight category prevalence estimation, following the recommendation to use national data when available.^49^ However, this may limit the generalizability of findings to non-German populations.

## Conclusion

In summary, our analysis does not demonstrate evidence that an antenatal lifestyle intervention conferred long-term clinical effects on somatic growth or neurodevelopment in children up to the age of five. Future research should focus on whether tailored approaches that target at-risk women lead to long-term health benefits for mothers and offspring.

## Supplementary information

Table S1

Table S2

## Data Availability

The datasets used and analyzed during the current study are available from the corresponding author upon reasonable request.

## References

[1] Llewellyn, A., Simmonds, M., Owen, C. G. & Woolacott, N. Childhood obesity as a predictor of morbidity in adulthood: a systematic review and meta-analysis. *Obes. Rev.: Off. J. Int. Assoc. Study Obes.* **17**, 56–67 (2016). 10.1111/obr.12316 26440472

[2] Tsiros, M. D. et al. Health-related quality of life in obese children and adolescents. *Int. J. Obes.* **33**, 387–400 (2009). 10.1038/ijo.2009.42 19255583

[3] Puhl, R. M. & King, K. M. Weight discrimination and bullying. *Best. Pract. Res. Clin. Endocrinol. Metab.* **27**, 117–127 (2013). 23731874 10.1016/j.beem.2012.12.002

[4] Heslehurst, N. et al. The association between maternal body mass index and child obesity: A systematic review and meta-analysis. *PLoS Med.* **16**, e1002817 (2019). 31185012 10.1371/journal.pmed.1002817 PMC6559702

[5] Voerman, E. et al. Maternal body mass index, gestational weight gain, and the risk of overweight and obesity across childhood: An individual participant data meta-analysis. *PLoS Med.* **16**, e1002744 (2019). 30742624 10.1371/journal.pmed.1002744 PMC6370184

[6] Fleming, T. P. et al. Origins of lifetime health around the time of conception: causes and consequences. *Lancet (Lond., Engl.)* **391**, 1842–1852 (2018). 10.1016/S0140-6736(18)30312-X PMC5975952 29673874

[7] Langley-Evans, S. C. Early life programming of health and disease: The long-term consequences of obesity in pregnancy. *J. Hum. Nutr. Dietetics : Off. J. Br. Dietetic Assoc.* **35**, 816–832 (2022). 10.1111/jhn.13023 PMC9540012 35475555

[8] Mehta, S. H., Kerver, J. M., Sokol, R. J., Keating, D. P. & Paneth, N. The association between maternal obesity and neurodevelopmental outcomes of offspring. *J. pediatrics* **165**, 891–896 (2014). 10.1016/j.jpeds.2014.07.003 25155965

[9] Rivera, H. M., Christiansen, K. J. & Sullivan, E. L. The role of maternal obesity in the risk of neuropsychiatric disorders. *Front. Neurosci.* **9**, 194 (2015). 26150767 10.3389/fnins.2015.00194 PMC4471351

[10] Navarro, P., Mehegan, J., Murrin, C. M., Kelleher, C. C. & Phillips, C. M. Associations between a maternal healthy lifestyle score and adverse offspring birth outcomes and childhood obesity in the Lifeways Cross-Generation Cohort Study. *Int. J. Obes. (2005)* **44**, 2213–2224 (2020). 10.1038/s41366-020-00652-x 32829383

[11] Arenz, S., Rückerl, R., Koletzko, B. & Kries, R. V. Breast-feeding and childhood obesity-a systematic review. *Int. J. Obes. Relat. Metab. Disord. : J. Int. Assoc. Study Obes.* **28**, 1247–1256 (2004). 10.1038/sj.ijo.0802758 15314625

[12] Georgieff, M. K., Ramel, S. E. & Cusick, S. E. Nutritional influences on brain development. *Acta paediatrica (Oslo, Nor. : 1992)* **107**, 1310–1321 (2018). 10.1111/apa.14287 PMC6045434 29468731

[13] Louise, J. et al. The effects of dietary and lifestyle interventions among pregnant women with overweight or obesity on early childhood outcomes: an individual participant data meta-analysis from randomised trials. *BMC Med.* **19**, 128 (2021). 34074261 10.1186/s12916-021-01995-6 PMC8170974

[14] Raab, R. et al. Associations between lifestyle interventions during pregnancy and childhood weight and growth: a systematic review and meta-analysis. *Int. J. Behav. Nutr. Phys. Act.* **18**, 8 (2021). 33413486 10.1186/s12966-020-01075-7 PMC7792105

[15] Rauh, K. et al. Healthy living in pregnancy: a cluster-randomized controlled trial to prevent excessive gestational weight gain - rationale and design of the GeliS study. *BMC pregnancy childbirth* **14**, 119 (2014). 24678761 10.1186/1471-2393-14-119 PMC3973835

[16] Hoffmann, J. et al. Effects of a lifestyle intervention in routine care on prenatal physical activity - findings from the cluster-randomised GeliS trial. *BMC pregnancy childbirth* **19**, 414 (2019). 31711430 10.1186/s12884-019-2553-7 PMC6849250

[17] Günther, J. et al. Effects of a Lifestyle Intervention in Routine Care on Prenatal Dietary Behavior-Findings from the Cluster-Randomized GeliS Trial. *J. Clin. Med.* **8**, 960 (2019). 10.3390/jcm8070960 PMC6678299 31269753

[18] Geyer, K. et al. Effects of a Prenatal Lifestyle Intervention in Routine Care on Maternal Health Behaviour in the First Year Postpartum-Secondary Findings of the Cluster-Randomised GeliS Trial. *Nutrients* **13**, 1310 (2021). 10.3390/nu13041310 PMC8071441 33921063

[19] Kunath, J. et al. Effects of a lifestyle intervention during pregnancy to prevent excessive gestational weight gain in routine care - the cluster-randomised GeliS trial. *BMC Med.* **17**, 5 (2019). 30636636 10.1186/s12916-018-1235-z PMC6330753

[20] Hoffmann, J. et al. Infant growth during the first year of life following a pregnancy lifestyle intervention in routine care-Findings from the cluster-randomised GeliS trial. *Pediatr. Obes.* **16**, e12705 (2021). 32725809 10.1111/ijpo.12705

[21] Spies, M. et al. Child Anthropometrics and Neurodevelopment at 2 and 3 Years of Age Following an Antenatal Lifestyle Intervention in Routine Care-A Secondary Analysis from the Cluster-Randomised GeliS Trial. *J. Clin. Med.* **11**, 1688 (2022). 10.3390/jcm11061688 PMC9040717 35330013

[22] Rasmussen, K. M. & Yaktine, A. L. Weight Gain During Pregnancy: Reexamining the Guidelines (2009). 20669500

[23] Hoffmann, J. et al. Effects of a Lifestyle Intervention in Routine Care on Short- and Long-Term Maternal Weight Retention and Breastfeeding Behavior-12 Months Follow-up of the Cluster-Randomized GeliS Trial. *J Clin. Med.* **8**, 876 (2019). 10.3390/jcm8060876 PMC6616390 31248138

[24] Koletzko, B. et al. Ernährung in der Schwangerschaft - Teil 2. Handlungsempfehlungen des Netzwerks “Gesund ins Leben - Netzwerk Junge Familie”. *Dtsch. medizinische Wochenschr. (1946)* **137**, 1366–1372 (2012). 10.1055/s-0032-1305076 22692838

[25] The American College of Obstetricians and Gynecologists ACOG Committee Opinion No. 650: Physical Activity and Exercise During Pregnancy and the Postpartum Period. *Obstet Gynecol.* **126**, e135–e142 (2015). 10.1097/AOG.0000000000001214 26595585

[26] Kromeyer-Hauschild, K. et al. Perzentile für den Body-mass-Index für das Kindes- und Jugendalter unter Heranziehung verschiedener deutscher Stichproben. *Monatsschr Kinderheilkd.* **149**, 807–818 (2001).

[27] Squires, J., Twombly, E., Bricker, D. & Potter, L. *ASQ-3™ User’s Guide* 3rd edn. (Paul H. Brookes Publishing, Baltimore, MD, USA, 2009).

[28] Bell, M. L., Kenward, M. G., Fairclough, D. L. & Horton, N. J. Differential dropout and bias in randomised controlled trials: when it matters and when it may not. *BMJ* **346**, e8668 (2013). 23338004 10.1136/bmj.e8668 PMC4688419

[29] Rauh, K. et al. Safety and efficacy of a lifestyle intervention for pregnant women to prevent excessive maternal weight gain: a cluster-randomized controlled trial. *BMC Pregnancy Childbirth* **13**, 151 (2013). 23865624 10.1186/1471-2393-13-151 PMC3718707

[30] Donner, A. & Klar, N. *Design and analysis of cluster randomization trials in health research* (John Wiley & Sons, Chichester, 2000).

[31] Braeken, M. A. K. A. & Bogaerts, A. Effect of Lifestyle Interventions in Obese Pregnant Women on the Neurocognitive Development and Anthropometrics of Preschool Children. *Obes. facts* **13**, 256–266 (2020). 32268328 10.1159/000506690 PMC7250361

[32] Teede, H. J. et al. Association of Antenatal Diet and Physical Activity-Based Interventions With Gestational Weight Gain and Pregnancy Outcomes: A Systematic Review and Meta-analysis. *JAMA Intern. Med.* **182**, 106–114 (2022). 34928300 10.1001/jamainternmed.2021.6373 PMC8689430

[33] International Weight Management in Pregnancy Collaborative Group (iWIP). Effect of diet and physical activity based interventions in pregnancy on gestational weight gain and pregnancy outcomes: meta-analysis of individual participant data from randomised trials. *BMJ* **358**, j3119 (2017). 28724518 10.1136/bmj.j3119 PMC6887834

[34] Dodd, J. M. et al. Effects of an antenatal dietary intervention in overweight and obese women on 6 month infant outcomes: follow-up from the LIMIT randomised trial. *Int. J. Obes. (2005)* **42**, 1326–1335 (2018). 10.1038/s41366-018-0019-z PMC6054603 29568100

[35] Dodd, J. M. et al. Prenatal Diet and Child Growth at 18 Months. *Pediatrics* **142**, e20180035 (2018). 10.1542/peds.2018-0035 30089655

[36] Dodd, J. M., Deussen, A. R. & Louise, J. Effects of an antenatal dietary intervention in women with obesity or overweight on child outcomes at 3-5 years of age: LIMIT randomised trial follow-up. *Int. J. Obes. (2005)* **44**, 1531–1535 (2020). 10.1038/s41366-020-0560-4 32203109

[37] Dodd, J. M., Deussen, A. R., Peña, A. S., Mitchell, M. & Louise, J. Effects of an antenatal dietary intervention in women with obesity or overweight on child outcomes at 8-10 years of age: LIMIT randomised trial follow-up. *BMC Pediatrics* **23**, 643 (2023). 38114910 10.1186/s12887-023-04466-4 PMC10729523

[38] Dalrymple, K. V. et al. Adiposity and cardiovascular outcomes in three-year-old children of participants in UPBEAT, an RCT of a complex intervention in pregnant women with obesity. *Pediatr. Obes.* **16**, e12725 (2021). 32914569 10.1111/ijpo.12725 PMC7116719

[39] Ong, K. K. & Loos, R. J. F. Rapid infancy weight gain and subsequent obesity: systematic reviews and hopeful suggestions. *Acta Paediatrica (Oslo, Nor. : 1992)* **95**, 904–908 (2006). 10.1080/08035250600719754 16882560

[40] Reilly, J. J. et al. Early life risk factors for obesity in childhood: cohort study. *BMJ* **330**, 1357 (2005). 15908441 10.1136/bmj.38470.670903.E0 PMC558282

[41] Menting, M. D. et al. Effects of maternal lifestyle interventions on child neurobehavioral development: Follow-up of randomized controlled trials. *Scand. J. Psychol.* **60**, 548–558 (2019). 31498898 10.1111/sjop.12575 PMC6899471

[42] Olmedo-Requena, R. et al. Variations in long-term outcome reporting among offspring followed up after lifestyle interventions in pregnancy: a systematic review. *J. Perinat. Med.* **48**, 89–95 (2020). 31926098 10.1515/jpm-2019-0302

[43] Raab, R., Geyer, K., Zagar, S. & Hauner, H. App-Supported Lifestyle Interventions in Pregnancy to Manage Gestational Weight Gain and Prevent Gestational Diabetes: Scoping Review. *J. Med. Internet Res.* **25**, e48853 (2023). 37948111 10.2196/48853 PMC10674147

[44] Geyer, K., Raab, R., Hoffmann, J. & Hauner, H. Development and validation of a screening questionnaire for early identification of pregnant women at risk for excessive gestational weight gain. *BMC pregnancy childbirth* **23**, 249 (2023). 37055730 10.1186/s12884-023-05569-7 PMC10100402

[45] Flynn, A. C. et al. Preventing and treating childhood overweight and obesity in children up to 5 years old: A systematic review by intervention setting. *Matern. child Nutr.* **18**, e13354 (2022). 35333450 10.1111/mcn.13354 PMC9218326

[46] Askie, L. M. et al. Interventions commenced by early infancy to prevent childhood obesity-The EPOCH Collaboration: An individual participant data prospective meta-analysis of four randomized controlled trials. *Pediatr. Obes.* **15**, e12618 (2020). 32026653 10.1111/ijpo.12618

[47] Geserick, M. et al. Acceleration of BMI in Early Childhood and Risk of Sustained Obesity. *N. Engl. J. Med.* **379**, 1303–1312 (2018). 30281992 10.1056/NEJMoa1803527

[48] Rolland-Cachera, M. F., Deheeger, M., Maillot, M. & Bellisle, F. Early adiposity rebound: causes and consequences for obesity in children and adults. *Int. J. Obes. (2005)* **30**, S11–S17 (2006). 10.1038/sj.ijo.0803514 17133230

[49] Rosario, A. S., Kurth, B.-M., Stolzenberg, H., Ellert, U. & Neuhauser, H. Body mass index percentiles for children and adolescents in Germany based on a nationally representative sample (KiGGS 2003-2006). *Eur. J. Clin. Nutr.* **64**, 341–349 (2010). 20179728 10.1038/ejcn.2010.8

