# Supplementary material for: Child somatic growth and neurodevelopment: effects of pregnancy lifestyle intervention
Source: Pediatr Res. 2025 Feb 20;98(2):585–92. doi: 10.1038/s41390-025-03936-y (PMC12454140; doi:10.1038/s41390-025-03936-y)
Supplement: Supplementary file 1 — Table S1 [file 41390_2025_3936_MOESM1_ESM.pdf]

Table S1 Statistical testing of between-group differences in characteristics of mother-child pairs.

	Intervention group n=712	Control group n=691	Total n=1403	*p* value [a]
**Maternal characteristics**				
Pre-pregnancy age, years [b]	30.6 ± 4.1	30.8 ± 4.3	30.7 ± 4.2	0.323
Pre-pregnancy weight, kg	67.9 ± 12.7	67.4 ± 13.1	67.6 ± 12.9	0.226
Pre-pregnancy BMI, kg/m^2	24.2 ± 4.2	24.0 ± 4.5	24.1 ± 4.3	0.132
Pre-pregnancy BMI category, n (%)				0.358
BMI 18.5–24.9 kg/m^2	467/712 (65.6%)	473/691 (68.5%)	940/1403 (67.0%)	
BMI 25.0–29.9 kg/m^2	170/712 (23.9%)	143/691 (20.7%)	313/1403 (22.3%)	
BMI 30.0–40.0 kg/m^2	75/712 (10.5%)	75/691 (10.9%)	150/1403 (10.7%)	
GWG, kg	13.8 ± 5.2	14.0 ± 5.0	13.9 ± 5.1	0.639
GDM, n (%)	72/700 (10.3%)	56/653 (8.6%)	128/1353 (9.5%)	0.283
Educational level, n (%) [c]				0.203
General secondary school	77/711 (10.8%)	96/690 (13.9%)	173/1401 (12.3%)	
Intermediate secondary school	307/711 (43.2%)	282/690 (40.9%)	589/1401 (42.0%)	
High school	327/711 (46.0%)	312/690 (45.2%)	639/1401 (45.5%)	
Country of birth, n (%)				0.473
Germany	640/712 (89.9%)	628/690 (91.0%)	1268/1402 (90.4%)	
Others	72/712 (10.1%)	62/690 (9.0%)	134/1402 (9.6%)	
Primiparous, n (%)	463/712 (65.0%)	384/691 (55.6%)	847/1403 (60.4%)	0.004
Current smoker, n (%) [d]	93/645 (14.4%)	75/612 (12.3%)	168/1257 (13.4%)	0.260
**Infant characteristics at birth**				
Sex, n (%)				0.287
Male	360/712 (50.6%)	369/691 (53.4%)	729/1403 (52.0%)	
Female	352/712 (49.4%)	322/691 (46.6%)	674/1403 (48.0%)	
Preterm birth, n (%)	43/709 (6.1%)	42/691 (6.1%)	85/1400 (6.1%)	0.992
SGA, n (%)	60/709 (8.5%)	51/691 (7.4%)	111/1400 (7.9%)	0.454
LGA, n (%)	55/709 (7.8%)	53/691 (7.7%)	108/1400 (7.7%)	0.951
Birth weight > 4000 g, n (%)	62/711 (8.7%)	58/691 (8.4%)	120/1402 (8.6%)	0.827

Abbreviations: BMI: body mass index; GWG: gestational weight gain; GDM: gestational diabetes mellitus; SGA: small for gestational age (< 10th percentile); LGA: large for gestational age (> 90th percentile); SD: standard deviation.

[a] *p* value for differences in baseline characteristics between mother-child pairs in the intervention and control group; examined using the $\chi 2$ test for categorical variables and the Kruskal–Wallis test for continuous variables.

[b] Mean ± SD (all such values).

[c] General secondary school: General school, which is completed through year 9; Intermediate secondary school: Vocational secondary school, which is completed through year 10; High school: Academic high school, which is completed through year 12 or 13.

[d] Collected five years after birth.
