# Supplementary material for: Child somatic growth and neurodevelopment: effects of pregnancy lifestyle intervention
Source: Pediatr Res. 2025 Feb 20;98(2):585–92. doi: 10.1038/s41390-025-03936-y (PMC12454140; doi:10.1038/s41390-025-03936-y)
Supplement: Supplementary file 2 — Table S2 [file 41390_2025_3936_MOESM2_ESM.pdf]

Table S2 Characteristics of mother-child pairs entering the 5-year follow-up compared to pairs lost to follow-up.

	Mother-child pairs in the 5-year FU n=1 408	Lost to FU at 5 years n=239	*p* value [a]
**Group assignment**			0.753
Intervention group	715/1408 (50.8%)	124/239 (51.9%)	
Control group	693/1408 (49.2%)	115/239 (48.1%)	
**Maternal characteristics**			
Pre-pregnancy age, years [b]	30.7 ± 4.2	30.4 ± 4.6	0.457
Pre-pregnancy weight, kg	67.7 ± 13.0	70.7 ± 13.9	0.002
Pre-pregnancy BMI, kg/m^2	24.2 ± 4.4	25.1 ± 4.6	0.001
Pre-pregnancy BMI category, n (%)			0.007
BMI 18.5–24.9 kg/m^2	940/1408 (66.8%)	137/239 (57.3%)	
BMI 25.0–29.9 kg/m^2	315/1408 (22.4%)	62/239 (25.9%)	
BMI 30.0–40.0 kg/m^2	153/1408 (10.9%)	40/239 (16.7%)	
GWG, kg	13.9 ± 5.1	14.6 ± 5.1	0.018
GDM, n (%)	129/1358 (9.5%)	24/228 (10.5%)	0.627
Educational level, n (%) [c]			<0.001
General secondary school	175/1406 (12.4%)	50/239 (20.9%)	
Intermediate secondary school	591/1406 (42.0%)	104/239 (43.5%)	
High school	640/1406 (45.5%)	85/239 (35.6%)	
Country of birth, n (%)			0.043
Germany	1273/1407 (90.5%)	206/239 (86.2%)	
Others	134/1407 (9.5%)	33/239 13.8%)	
Primiparous, n (%)	850/1408 (60.4%)	122/239 (51.0%)	< 0.001
**Infant characteristics at birth**			
Sex, n (%)			0.122
Male	731/1408 (51.9%)	137/239 (57.3%)	
Female	677/1408 (48.1%)	102/239 (42.7%)	
Preterm birth, n (%)	85/1405 (6.0%)	13/239 (5.4%)	0.712
SGA, n (%)	112/1405 (8.0%)	19/239 (7.9%)	0.991
LGA, n (%)	108/1405 (7.7%)	15/239 (6.3%)	0.443
Birth weight > 4000 g, n (%)	120/1407 (8.5%)	19/239 (7.9%)	0.766

Abbreviations: FU: follow up; BMI: body mass index; GWG: gestational weight gain; GDM: gestational diabetes mellitus; SGA: small for gestational age (< 10th percentile); LGA: large for gestational age (> 90th percentile); SD: standard deviation.

[a] *p* value for differences between mother infant-pairs who completed the 5-year FU and those who were lost to FU at 5-year mark; examined using the $\chi 2$ test for categorical variables and the Kruskal–Wallis test for continuous variables.

[b] Mean ± SD (all such values).

[c] General secondary school: General school, which is completed through year 9; Intermediate secondary school: Vocational secondary school, which is completed through year 10; High school: Academic high school, which is completed through year 12 or 13.
